# Studies on agarolytic bacterial isolates from agricultural and industrial soil

**Published:** 2018-10

**Authors:** Shomini Parashar, Narendra Kumar

**Affiliations:** Department of Biotechnology, IMS Engineering College, Ghaziabad, Uttar Pradesh, India

**Keywords:** Agarolytic, Agarase, Relative activity, Mineral salt Agar, *Microbacterium*

## Abstract

**Background and Objectives:**

Soil is rich in microbes which can be used for a variety of purposes starting from decomposition to antibiotic production. Agar-agar, extracted from the marine environment, is an important polysaccharide that has multiple uses after degradation by microbes. The aim of this study was to isolate bacteria that produced agarase enzyme, from a variety of soil sources and study their morphological and biochemical characterization. The enzyme activity of the isolates was also studied at 3 different pH, temperature and agar concentration.

**Materials and Methods:**

Agarolytic isolates, were identified from industrial and agar- enriched agriculture soil by serial dilution method using MSA media that contains agar as the only source of carbon. Qualitative analysis of the isolates was determined by iodine assay while for quantitative analysis of enzyme activity, at standard and variable conditions, DNSA method was used. Genus of SELA 4 was identified.

**Results:**

4 isolates were obtained from industrial soil and 6 were obtained from agriculture soil enriched with laboratory agar. Isolate ‘SELA 4’ showed maximum relative activity (OD 0.92) followed by ‘CCIL 2 (OD 0.91) under standard culture conditions. Isolate ‘SELA 1’ showed maximum activity between 37°C–40°C, pH 5–7 with 1.5% agar concentration. “CGIPL 1” showed maximum activity at pH 9 while “SELA 2” and “SELA 4” showed maximum activity at pH 5. SELA 4 belonged to genus *Microbacterium* (Accession no. MG203882.1).

**Conclusion:**

The results showed that agar degrading bacteria can also be isolated from soil sources other than the usual marine sources and can be used for the industrial production of agarase enzyme.

## INTRODUCTION

Several species of agarase enzyme producing bacteria, isolated from various sources, degrade agar by producing extra or intracellular enzymes (1, 2). As agar is the major component of red algae, it is more likely that agar degrading bacteria are isolated from marine sources but a few agar degrading bacterial isolates are also obtained from other sources like compost (3), plants (4), soil (5) are capable of utilizing agar as the only source of carbon and energy. Among the reported agar-degrading bacteria from various soil source, many different genera have been found as shown in Table 1. Soil acts as a habitat for a wide variety of micro-organisms as compared to other environments. Thus, it was chosen as a source for isolating agarolytic bacteria in the present study. Agar is a polymer made up of galactose sugar subunits and occurs in the cell wall of algae, mainly *Gelidium, Gracilaria* as structural carbohydrate and provide primary structural support to it in the form of its calcium salt or a mixture of their calcium and magnesium salts. Agar is constituted of two major parts; Agarose (70%), the gelling fraction of agar and Agaropectin (30%), the non-gelling fraction of agar (6). Agar is generally used to solidify growth media for culturing microorganisms. Agar is firmer and stronger and is not easily degraded by most bacteria. Those that degrade it, has three ways of degradation: The first way is disruption of the double helical structure without the breakdown of the polymer. This limited activity does not produce any visible changes on the agar but can be detected when iodine fails to form the dark brown color with the gel (7). The second way is breaking down of the α-linkages of the agar molecule which give rise to oligosaccharides of the agarobiose series with 3, 6-anhydroL-galactose at the reducing end (8). The third way is breaking down of the β-linkages which give rise to the neo- agarobiose series of oligosaccharides with D-galactose at the reducing end (2). The enzymes involved in the second and third categories are called α- agarases (*E.C. 3.2.1.158*) and β-agarases (*E.C. 3.2.1.81*) respectively.

In our study, we isolated agarolytic bacteria from industrial and agriculture soil enriched regularly by non-pathogenic laboratory nutrient agar waste and obtained the pure culture of the ten isolates, namely CCIL 1, CCIL 2, CGIPL 1, CGIPL 2, SELA 1, SELA 2, SELA 3, SELA 4, SELA 5, and SELA 6. Only four isolates were obtained from industrial soil and six isolates were isolated from agriculture soil which was enriched with discarded and decanted laboratory agar. After obtaining the cultures in pure form, their morphology, growth characteristics, growth pattern, biochemical properties were studied and culture conditions were optimized for the production of agarase enzymes.

**Table 1.** Table of bacterial isolates from different soil sources

**Bacteria**	**Source**	**Gram staining**	**Enzyme Secretion**	**Enzyme type/name**	**Isolation Media used**	**Mr (kDa)**	**Specific activity for agar (U/mg)**	**Optimal T (°C)**	**Stable up to T (°C)**	**Optimal pH**	**Stable pH**	**Product type**	**Ref.**
*Ammonibacillus agrariperforans* FAB2 (T)	Sewage sludge compost	G+ve Aerobic	-	Agarase	B2A Agar	-	-	60	50–65	8.0–8.6	7.5–9.0	-	(3)
*Endophytic Bacteria* (7 strains)	Plant		Extracellular	Agarase	Mineral salt Agar	75 kDa	-	37	-	7.5	-	-	(4)
*Paenibacillus sp.* SSG-1	Soil	G +ve	Extracellular	β-agarases SSG-1a, 1b, 1c	Mineral salt agar	77 kDa	111	50	40	6	4.0–10.0	NA4, NA6	(5)
*Steroidobacter agriperforans sp.* KA5-BT	Soil	G −ve	-	-	PSG medium	-	-	30	15–37	6.0–8.0	4.5–9.0	-	(9)
*Bacillus sp*., H12	Greenhouse soil	G +ve	-	-	Nutrient agar	-	48.5 (Ion) 68.09 (Gel)	45	35–45	7	4.0–10.0	-	(10)
*Streptomyces coelicolor* A3 (2)	Soil	G +ve	Extracellular	β-agarases-II SCO3487	R2YE agar	88.5 kDa	0.57	40	20 to 70	7	5.0–11.0	NA4, NA6	(11)
*Simiduia areninigrae sp.* Nov (M2-5T)	Black sand	G −ve	-	β-agarases, catalase, oxidase	MA plates or PY	-	-	25–30	10–37	8.0–9.0	7.0–9.0	-	(12)
*Acinetobacter sp.* AGLSL-1	Soil	G −ve	Extracellular	Czapek-β-agarases	Dox Agar	100	397	40	45	6	5.0–9.0	NA2	(13)
*Alteromonas sp.* SY37-12	Marine algae	G −ve	Extracellular	β-agarases		39.5	83.5	35	50	7	-	NA4, NA6 (main), NA8	(14)
*Asticcacaulis sp.* SA7	Rhizosphere soil	G −ve	Extracellular	-	Basal media	70 kDa	-	28	-	6.2	-	-	(15)
*Thalassomonas sp.* JAMB-A33	Marine sediment	G −ve	Intracellular	α-agarases	-	85	40.7	45	40	8.5	6–11	A2, A4 (main), A6	(16)
*Bacillus sp.* MK03	Soil	G +ve	Intracellular	β-agarases	Nutrient agar	92 (SDS-PAGE)	14.2	40	35	7.6	7.1–8.2	NA2, NA4 (main)	(2)
*Paenibacillus sp.* (M-2b, O-3b, O-4c, St-4)	Rhizosphere of Spinach	G +ve	Extracellular	β-agarases	TSA agar	75–160 kDa	-	28	50–45	7	-	-	(17)
*Vibrio sp*. LX-3	Soil	G −ve	Extracellular	Car-boxymethyl cellulase CMC	Mineral salt Agar	-	33.2 U/l	30	5.0–37	7.0–7.25	5.5–10.5	agaro-oligosac- charides	(18)

## MATERIALS AND METHODS

### Isolation of agar degrading bacteria.

For the isolation of agarolytic bacteria, soil samples were collected from various sources of Ghaziabad region namely IMS Engineering College campus, Bharat Chemical Products Pvt. Ltd. (BCPPL), Continental Carbon India Ltd. (CCIL), Crop Growth India Pvt. Ltd. (CGIPL), Agriculture Soil Enriched with Laboratory Agar (SELA). A small amount of soil was collected from each source and serially diluted by 10 fold serial dilution method (10^−1^, 10^−2^, 10^−3^, 10^−4^, 10^−5^ and 10^−6^). Suspension of the soil sample was made by dissolving 1gm of soil from the source in 100 ml of normal saline. Three replica plating was done from the suspension taken from dilution tubes 10^−4^, 10^−5^, 10^−6^. 100 μl of suspension was spread on petriplates which contained 1.5% agar in MSA (Mineral Salt Agar) medium composed of (g/l) CaCl_2_ 0.1; MgSO_4_ 0.5; (NH4) 2SO_4_ 0.5; K2HPO_4_ 0.5; NaCl 0.5 with 50 mg/l Nystatin (to avoid fungal growth). The pH of the media was adjusted to 7.0 and the plates were incubated at 37°C for 24 hours. Colonies which showed depression on the agar plate were selected and pure culture was obtained after 3–4 consecutive sub-culturing on to fresh media plates by quadrant streaking procedure. Growth was observed after 24 hours but depression on an agar plate could be observed after 48 hours of incubation. A total of 2, 2 and 6 colonies having depression were picked from CCIL, CGIPL and SELA soil sample plates respectively. The colonies were carefully examined and differentiated based on colony characteristics. The bacterial cultures were designated as CCIL1 & 2, CGIPL1 & 2, and SELA 1–6. The isolated cultures were maintained at 4°C on MSA slants having agar as the only source of carbon and energy. The plates were sub-cultured fortnightly.

### Morphological and biochemical characteristics of the isolates.

The isolates after 3 days were observed for their colony morphology like margins, elevation, texture/consistency, colony surface, color, opacity on the solid agar plates. Simple staining was done by flooding the dried smear slide with crystal violet for a minute. Gram staining was done on the slide having air-dried smear of bacteria, as described by Murray et al. (1994) (19). Capsular staining of the bacterial culture was done by staining the slide for 3 minutes with 1% (wt/vol) aqueous solution of crystal violet and flooding the slide with 20% (wt/vol) CuSO_4_ solution. Biochemical tests for Catalase, Indole (20), Methyl red (21) and Citrate utilization (22) for the isolates were also done in the process of identification.

### Qualitative and Quantitative assays.

In order to detect the presence of agarase enzyme in the isolated bacterial cultures, qualitative and quantitative tests were performed. For the qualitative test, the culture plates were flooded with Lugol’s iodine solution (1g Iodine, 2g KI, 100 ml distilled water) which stains polysaccharide of agar into dark brown color while it cannot stain degraded oligosaccharide of agar. For the quantitative assay, DNSA method (23) was used. In this test, the enzyme activity was measured by the release of the reducing sugar equivalent using DNSA (3,5- di-nitrosalicylic acid) and spectrophotometrically increasing concentration of reducing sugar was determined keeping D-galactose as standard.

### Partial purification of agarase enzyme.

Each bacterial isolates (10) were inoculated into MSA broth supplemented with 0.3% (w/v) agar. The flasks containing 50 ml of culture media inoculated with 1 ml of culture in its log phase were incubated on a rotary shaker at 170 rev min^−1^ for 24 hours at 37°C. The cultures were centrifuged in the centrifuge tubes at 10,000 rev min^−1^ for 10 min. at 4°C. The supernatant obtained was used as a source of agarase enzyme for testing the activity.

### Detection of agarase activity.

The enzyme activity was detected using DNSA method (23). The Stock solution for galactose was prepared by taking 250 mg of galactose in 100 ml of distilled water. From this stock solution, a working solution was prepared by adding 9 ml of distilled water in 1 ml of this stock solution. In 7 clean, dry test tubes, the standard sugar solution was pipetted out in the range of 0 to 3 ml in different test tubes and the volume of all test tubes was made to 3 mL with distilled water. Finally, each tube was added with 1 ml DNS reagent and by putting the cotton plug, the test tubes were kept in boiling water bath for 5 minutes. After cooling the tubes at room temperature, optical density was measured at 540 nm against the blank. The values for reducing sugars were expressed as galactose equivalents. For measuring the enzyme activity, 100 μl sample of enzyme supernatant was mixed with 900 μl Phosphate Buffer Saline (pH, 7.0) with 0.25% agar substrate and incubated at 37°C for 15 minutes. Initial OD was recorded. Then 1 ml of this reaction mixture was added with 2 ml of DNSA reagent and heated for 5 min at 100°C in the water bath. After cooling, the mixture is diluted with 10 ml of distilled water and optical density reading was recorded at 540 nm.

The enzyme activity was measured in Units per ml and one unit of agarase activity was defined as the amount of enzyme that released 1μmol reducing sugar (measured as galactose) from agar per minute under defined conditions (24).

### Effect of physical parameters on growth and production of agarase.

The physical parameters of all the isolates were determined after 3 days of incubation at 37°C. The effect of temperature, pH and agar concentration, on the growth and production of agarase, was studied. The temperature variations taken were 28°C, 37°C and 40°C. The pH variations taken were 5, 7 and 9 while the agar concentrations taken were 0.05%, 0.1% and 1.5% agar. The growth of bacteria was measured by spectrophotometer at 540 nm and the enzyme activity was also recorded simultaneously as described above. The activity was plotted graphically to determine the optimal growth of the isolated bacteria.

## RESULTS

### Isolation of Agar degrading bacteria.

Ten morphologically different agar degrading bacterial isolates were obtained from the soil sample by serial dilution method, from 6 different sites. Out of fifty-four plates, only five plates showed depression/slight liquefaction on the agar plates. From these five plates, 10 morphologically distinct colonies, which produced depression around them, were picked and pure cultures were obtained by quadrant streaking. The isolates were named after the source of isolation as SELA 1, SELA 2, SELA 3, SELA 4, SELA 5, SELA 6, CGIPL 1, CGIPL 2, CCIL 1, CCIL 2. For convenience they were named from A to J. The cultures are maintained on MSA slants with 1.5% agar (w/v) at 4°C and are sub-cultured fortnightly.

### Morphological and biochemical characteristics of the isolates.

In general, the isolates obtained, formed round colonies with a smooth texture and flat surface. The colony color ranged from cream to pale yellow (Table 2).

**Table 2.** Colony morphology of the bacterial isolates showing depression on agar

**Isolate**	**Shape**	**Margin**	**Elevation**	**Texture**	**Colour**	**Special features**
A (SELA 1)	Round	Entire	Flat	Smooth	Off-white	Depression has shown on the 4th day of incubation
B (SELA 2)	Round	Entire	Flat	Smooth	Shiny Cream	Dark color center with light borders. Small colonies
C (CGIPL 1)	Round	Entire	Flat	Smooth	Pale yellow	Dark color center with light borders. Small colonies
D (SELA 5)	Round	Entire	Flat	Smooth	Shiny Cream	Clear zone and slight depression is seen after 24 hours
E (CGIPL 2)	Round	Entire	Flat	Smooth	Off-white	Colonies are large sized with a defined center and light periphery
F (CCIL 1)	Round	Lobate	Flat	Smooth	Pale yellow	Clear zones and depression is seen after 24 hours
G (SELA 3)	Round	Entire	Flat	Smooth	Cream	Slight depression after 24 hours which increased after 48 hours
H (SELA 4)	Round	Entire	Flat	Smooth	Off-white	Marked zone of clearance
I (SELA 6)	Irregular	Lobate	Flat	Smooth	Cream	Flower-like colony with a defined center
J (CCIL 2)	Irregular	Lobate	Flat	Smooth	Pale yellow	Clear zones have seen after 24 hours

According to Table 3, the physiological and biochemical characteristics of the isolates showed that all except SELA 1 and SELA 6 were Gram-positive. All isolates were capsular negative and were rod-shaped except SELA 6. None of the isolates decomposed amino acid tryptophan to indole. All were catalase negative except CCIL 2. Only CCIL 1 and SELA 3 utilized citrate (tryptophan) as a source of energy.

**Table 3.** Physiological and biochemical properties of the bacterial isolates (A–J)

**Bacterial**	**Staining**			**Biochemical tests**				

**Isolates**	**Simple**	**Gram**	**Capsular**	**Iodine test**	**Methyl red**	**Citrate**	**Indole**	**Catalase**
A (SELA 1)	Rod shape	G −	−	+	−	−	−	−
B (SELA 2)	Rod shape	G +	−	+	−	−	−	−
C (CGIPL 1)	Rod shape	G +	−	+	−	−	−	−
D (SELA 5)	Rod shape	G +	−	+	−	−	−	−
E (CGIPL 2)	Rod shape	G +	−	+	+	−	−	−
F (CCIL 1)	Rod shape	G +	−	+	−	+	−	−
G (SELA 3)	Rod shape	G +	−	+	+	+	−	−
H (SELA 4)	Rod shape	G +	−	+	+	−	−	−
I (SELA 6)	Cocci	G −	−	+	+	−	−	−
J (CCIL 2)	Rod shape	G +	−	+	−	−	−	+

### Qualitative assays.

**Fig. 1.**
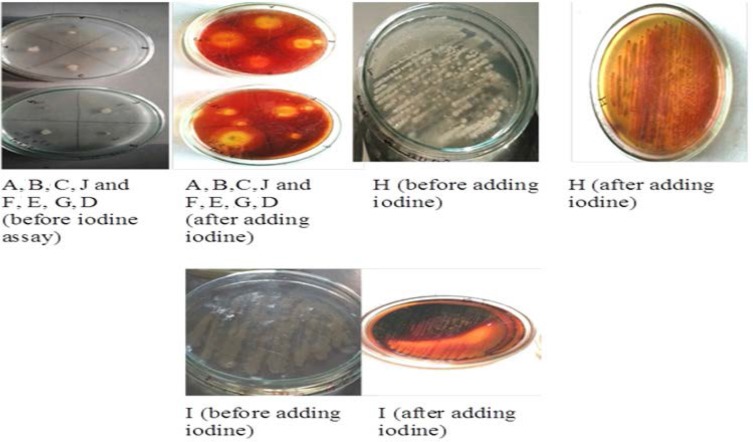
Iodine assay for agarolytic bacteria (Qualitative assay)

The iodine assay for all the bacterial isolates was performed. All the isolates showed a clear light color zone around the culture when dark brown color iodine was poured on the culture plates (Fig. 1).

### Determination of agarase activity.

**Fig. 2.**
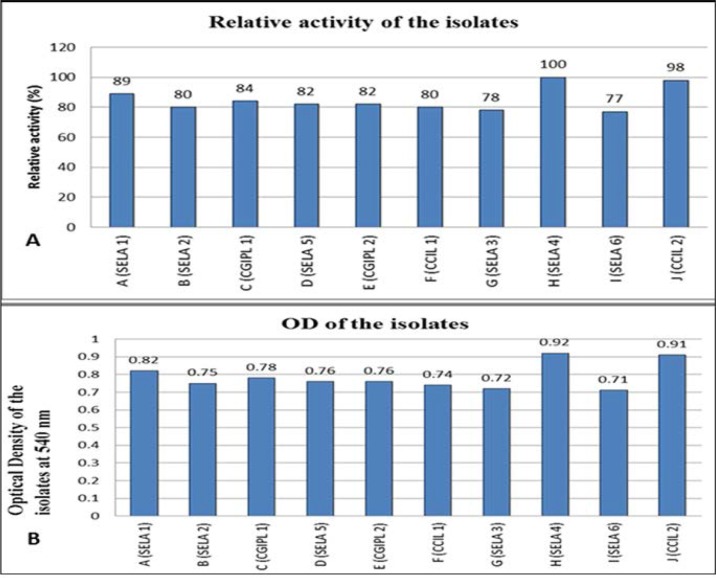
(A) The figure shows the relative activity of all the isolates at standard conditions. The isolate with maximum activity was taken as 100% and the relative activity of all the cultures was plotted with reference to the one with maximum activity (SELA 4) (B) The figure shows OD of the isolates at a wavelength 540 nm

Fig. 2. shows the relative activity of all the isolates at standard conditions. The isolate with maximum activity was taken as 100% and the relative activity of all the other isolates was plotted with reference to the one with maximum activity (SELA 4). It demonstrates that the SELA 4 shows the highest relative activity while the SELA 6 showed the least relative activity at 37°C. The decreasing order of the activity is SELA 4>CCIL 2>SELA 1>CGIPL 1>SELA 5, CGIPL 2>SELA 2, CCIL1>SELA 3>SELA 6.

### Effects of temperature on agarase activity.

**Fig. 3.**
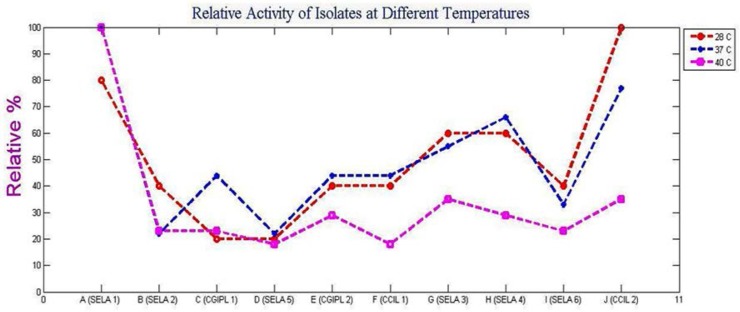
Relative activity of the isolates at different temperature

Fig. 3 shows the optimal temperatures of the isolates for agarase activity. At 28°C, maximum activity was shown by CCIL 2 and then it decreases from CCIL 2 to SELA 1> SELA 3, SELA 4 >SELA 2, CGIPL 2, CCIL 1, SELA 6> CGPIL 1, SELA 5. At 37°C the maximum activity is shown by SELA 1 followed by CCIL 2 >SELA 4>SELA 3> CCIL 1, CGPIL 2, CCIL 1>SELA 6>SELA 2, SELA 5. Also when the isolates were incubated at 40°C for 24 hours, the activity in decreasing order was shown by culture SELA 1> SELA 3, CCIL 2> CGIPL 2, SELA 4> SELA 2, CGPIL 1, SELA 6>SELA 5 and CCIL 1. The isolates showed relatively very low activity at 40°C.

### Effects of pH on agarase activity.

**Fig. 4.**
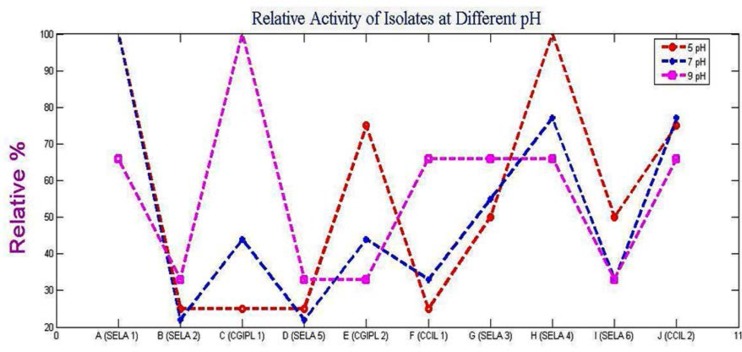
Relative activity of the isolates at different pH

Fig. 4 represents the effect of pH on the activity of a partially purified crude extract of all the 10 isolates. The graph shows high fluctuation and variations at different pH. At pH5, maximum activity was shown by SELA 1 and SELA 4 followed by CGIPL 2> SELA 3, SELA 6> SELA 1, SELA 2, SELA 5, CGIPL 1. At pH 7 also the culture SELA 1 showed maximum activity while all the other cultures showed moderate or low activities in the order SELA 1> SELA 4, CCIL 2>SELA 3>CGIPL 1, CGIPL 2> CCIL1> SELA 6>SELA 2 and SELA 5 which showed negligible activity. However, the maximum activity at pH 9 was shown by the culture CGIPL 1 followed by SELA 1, SELA 3, SELA 4, CCIL 1, CCIL 2 > SELA 2, SELA 5, SELA 6, CGIPL 2.

### Effects of agar concentration on agarase activity.

**Fig. 5.**
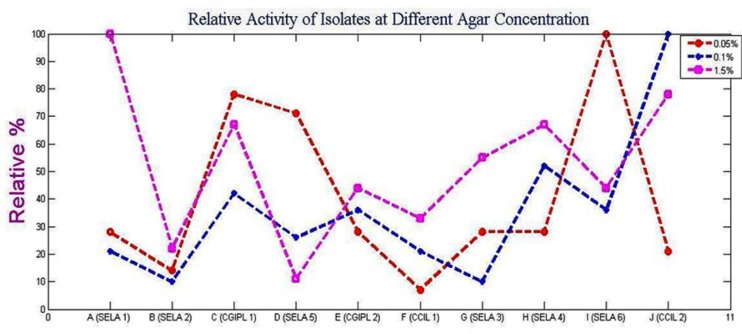
Relative activity of the isolates at different agar concentration

Fig. 5. showed the effect of different agar concentration on the enzyme activity of the bacterial isolates. It was seen that with 1.5% agar in the culture media, the isolate SELA 1 showed maximum activity which was followed by CCIL 2> CGIPL 1, SELA 4> SELA 3> CGIPL 2, SELA 6>CCIL 1> SELA 2>SELA 5. At 0.1% agar concentration, maximum activity was shown by culture ‘CCIL 2’ followed by SELA 4> CGIPL 1>CGIPL 2, SELA 6>SELA 5> CCIL 1, SELA 1> SELA 2, SELA 3 while at 0.05% agar ‘SELA 6’ showed maximum activity and ‘CCIL 1’ showed the least activity. The activity decreased from SELA 6>CGIPL 1>SELA 5>SELA 3, SELA 4, SELA 1, CGIPL 2>CCIL 2>SELA 2> CCIL 1. Figs 3, 4, 5 shows the graph of the relative activity of all the 10 isolates, where the maximum enzyme activity was defined as 100% and the relative activity is defined as the actual activity/maximum activity × 100.

## DISCUSSION

All the soil samples after serial dilution were spread on Mineral Salt Agar media for screening the agarolytic bacteria. When samples were plated onto the Mineral Salt Agar medium, the gradual degradation of agar was seen. Depressions were formed around the colonies which indicate agar degradation (Fig. 1). When the iodine solution was poured over the plates, pale yellow zones around the colonies were observed (Fig. 2). The observed results were compared with other agar-degrading bacterial species. The bacteria isolated from non-marine sources generally grow at a temperature ranging from 30°C–60°C with few exceptions. In the present study, some of the isolated cultures grow at temperature ranges between 35°–37°C similar to the seven endophytic bacterial strains (4) and *Alteromonas sp.* SY37-12 (14). While the culture, ‘SELA 1’ has shown good activity at 40°C similar to many *Bacillus sp*. (2), *Alteromonas sp*. E1 (25), *Vibrio* (26), *Acinetobacter sp*. AGLSL-1 (13), *Streptomyces coelicolor* A3 (2) (11) which were also isolated from soil sources. It is also seen that as temperature decreases; the activity of the enzyme also decreases (Fig. 4) The cultures ‘CCIL 2’, ‘CGIPL 1’, and ‘SELA 6’ showed optimal growth at 28°C temperature like *Simiduiaareninigrae sp*. Nov (M2-5T) isolated from black sand (12), *Asticcacaulis sp*. SA7 (15), isolated from rhizosphere soil, *Paenibacillus sp.* (17) isolated from the rhizosphere of spinach which also grows well at 28°C. The agarase enzyme produced by seawater-derived bacteria, i.e., *Vibrio sp.* JT0107 and *Alteromonas sp.* C-1, possess the optimal activity at 30°C (27, 28). Because these are isolated from the marine environment, they are not stable at high temperature. Only agarase enzyme isolated from *Alteromonas sp*. SY37-12 and *Agarivorans* YKW-34 have been found to be stable up to 50°C, others are stable up to 45°C (29, 30), 40°C (27, 16), 30°C (28, 31, 32) and 25°C (33). According to the report of Lakshmikanth et al. (13), most of the agarolytic bacteria grow at a pH range from 6.5–7.8. *Pseudomonas aeruginosa* grow at the broad pH range of 5.0–11. Hosoya et al. (2009) reported *Psychromonas agarivorans* to be slightly alkali tolerant with pH growth range of 6 to 9 and with an optimum pH requirement of 8 to 9. *Bacillus subtilis* as an alkali-tolerant has a specific requirement of sodium chloride for growth and production of extracellular agarases just like most of the agarolytic bacteria isolated from marine sources (34). Most of the agarase enzyme from the marine environment have been reported to show their maximum activity at a neutral pH (14, 31, 32) or at week alkaline pH (29, 27, 16, 33). While the agarase isolated from *Vibrio sp.* AP-2 and *Alteromonas sp.* C-1 has been shown their maximum activity at pH 5.5 (30) and pH 6.5 (28), respectively. The current study states that the isolated culture, ‘SELA 1’ showed maximum activity at pH between 5–7 but its activity decreased gradually when the temperature decreases from 40°C to 28°C (Fig. 3) and when the pH increases (Fig. 4). It is also evident from the graph in Fig. 5 that the isolated cultures require 1.5% agar concentrations for optimal enzyme activity. However, the culture ‘CCIL 2’ showed good activity at 37°C, pH 7 and at 0.1% agar concentration. All the cultures showed increased enzyme activity when agar concentration was increased from 0.10% to 1.50% except ‘SELA 5′ and ‘CCIL 2’ which showed the highest activity at 0.05% and 0.1% agar concentration respectively.

## CONCLUSION

The current work focused on the isolation of agar degrading bacteria from industrial and agriculture soil enriched with laboratory agar. A majority of cultures (n=6) were obtained from soil enriched with laboratory agar while four isolates were obtained from industrial soil. Eight isolates were Gram-positive and two were Gram-negative in nature. The isolates grew well in MSA (Mineral Salt Agar) media. All the isolates grown on solid media were aerobic, showed clear bright zone around the colonies which degrade agar as shown by the iodine test and showed pits on the surface of the agar medium. It is concluded that the maximum enzyme activity was shown by the isolate ‘SELA 4’ and showed optimal activity at 37°C, pH 5 with 1.5% agar concentration. It was seen in the study that temperature and pH have a strong impact on the activity of the enzyme, agarase. Also when the composition of agar in media was altered, the activity of the enzyme is also altered. Most of the soil isolates were Gram-positive rods showing optimal growth at a moderate temperature, slightly acidic pH and high agar concentration. It is evident that agar degrading bacteria can not only be isolated from marine sources but also from other sources like soil.
